# Prevalence, risk factors, impact and management of constipation among adults in Urumqi, China: a cross-sectional survey

**DOI:** 10.3389/fnut.2024.1451527

**Published:** 2024-11-01

**Authors:** Lingyun Shi, Li Shi, Minghui Wei, Mengke Zhang, Yuan Zhang, Jiaxue Li, Palida Maimaiti

**Affiliations:** ^1^School of Nursing, Xinjiang Medical University, Urumqi, China; ^2^Xinjiang Medical University, Urumqi, China; ^3^The First Affiliated Hospital of Xinjiang Medical University, Urumqi, China

**Keywords:** constipation, prevalence, risk factors, dietary patterns, a cross-sectional survey

## Abstract

Constipation, a prevalent gastrointestinal disorder, significantly impacts quality of life. This study aimed to investigate the prevalence, risk factors, and implications of constipation in Urumqi City. A cross-sectional survey was conducted from July to August 2023, involving 1,386 participants aged 20–80 years. Data were collected via a self-administered questionnaire, with constipation severity assessed using the Patient Assessment of Constipation Symptoms (PAC-SYM) scale. Key findings indicated that various factors, including residing in rural areas, outdoor work, and sleep disorders, were positively correlated with higher PAC-SYM scores. Conversely, higher daily water intake and certain dietary patterns showed negative correlations. Additionally, increased constipation severity was associated with greater physiological strain and psychosocial concerns (all *p* < 0.05), emphasizing the disorder’s profound effect on quality of life. These findings underscore the need for comprehensive management strategies in Urumqi, considering its diverse lifestyle and dietary factors.

## Introduction

Constipation, a prevalent gastrointestinal disorder, plagues a significant fraction of the global populace. Manifesting as infrequent bowel movements, hardened or lumpy stools, and a lingering sensation of incomplete evacuation, this condition transcends mere physical discomfort. It considerably diminishes an individual’s quality of life, casting shadows on psychological and social facets of well-being (1). While recent studies report constipation prevalence rates ranging from 10.4 to 15.6% among adults in Western countries (2, 3), gastrointestinal symptoms, including constipation, remain particularly prevalent among adults in Chinese communities. Recent investigations indicate that nearly 18.6% of this demographic grapple with at least one such symptom, with constipation emerging as the predominant concern (4). Yet, data specific to regions, especially Xinjiang’s capital, Urumqi, remains conspicuously sparse.

The repercussions of chronic constipation are manifold, encompassing complications like hemorrhoids, anal fissures, and even graver conditions such as colorectal cancer (5, 6). The prolonged therapeutic journey it necessitates is not only cumbersome but also financially taxing. The economic ramifications of constipation are profound, stemming from both direct medical expenditures and productivity losses (7).

Urumqi, with its rich mosaic of ethnicities, presents a unique and valuable context for examining constipation. The city’s diverse population, encompassing a range of cultural and dietary practices, offers a distinctive lens through which the complex etiology of constipation can be explored. This diversity is particularly relevant given the potential influence of lifestyle and dietary habits on gastrointestinal health. Urumqi’s multi-ethnic composition, therefore, not only enhances the depth of our study but also provides an opportunity to uncover new insights into the risk factors and patterns associated with constipation.

In recognition of the profound impact of constipation on public health and the apparent lack of specific data within the Chinese context, this study aims to investigate the prevalence, determinants, and effects of constipation among the adult residents of Urumqi. The primary goal of this cross-sectional study is to systematically assess the prevalence and characteristics of constipation in the adult population of Urumqi, as well as to evaluate the correlation between the severity of constipation and their quality of life. In the process, we endeavor to identify a range of factors that may influence both the prevalence and severity of constipation – including dietary, lifestyle-related, and those associated with coexisting conditions. This comprehensive approach enables us to gain a deeper understanding of the multifaceted nature of constipation and its broader implications on the well-being of affected individuals.

## Participants

In this cross-sectional study conducted in Urumqi City, Xinjiang, China, a multi-stage stratified whole cluster sampling method was employed to gather a representative sample of 1,531 adults aged 20 and above, all of whom had resided in their current location for over a year. This sampling approach was meticulously aligned with the gender, age, and ethnic distribution data from the 2019 China National Population Census (CNA) specific to Urumqi City. The stratification criteria were based on the primary socio-demographic factors, including gender, age groups (20–35, 36–50, 51–65, 66–80 years), and ethnic composition (Han, Uyghur, Kazakh, Hui, and others).

The random selection at each stage was conducted using computer-generated random numbers to ensure impartiality. In the initial phase, four administrative districts in Urumqi City—Xinshi, Toutunhe, Tianshan, and Shayibake—were randomly selected. These districts were chosen to represent the varied socio-economic statuses and lifestyles prevalent in Urumqi’s six districts and one county. Next, within each of these selected districts, two communities were randomly chosen using random number generators. Finally, within each community, one neighborhood was randomly selected to participate in the survey.

This rigorous process ensured that the sample was representative of the population distribution in Urumqi. Of the 1,531 distributed questionnaires, 1,386 were considered valid, post exclusion of non-participants and those outside the age range, resulting in a response rate of 90.5%. The comprehensive selection process is illustrated in Supplementary Figure S1. Ethical approval for this study was granted by the research ethics committee of the First Affiliated Hospital of Xinjiang Medical University (Approval No.20180223–170), with informed consent obtained from all participating individuals.

### Investigation techniques

We utilized a bespoke questionnaire, segmented into six primary categories:

1. Demographic Data: Captured details such as gender, age, ethnicity, height, weight, residence, education, and occupation.
2. Patient Assessment of Constipation Symptoms (PAC-SYM) (8): The Patient Assessment of Constipation Symptoms (PAC-SYM) is a 12-item, self-reported questionnaire designed to evaluate the severity of constipation-related symptoms. The items are divided into three subscales, assessing abdominal symptoms (e.g., discomfort, bloating), rectal symptoms (e.g., painful defecation, straining), and stool symptoms (e.g., incomplete evacuation, hard stools). Each item is scored on a 5-point Likert scale, where 0 indicates “absent” and 4 indicates “very severe.” The total PAC-SYM score is calculated by averaging the item scores, with higher scores indicating greater symptom severity. The scale has demonstrated high internal consistency (Cronbach’s *α* = 0.89) and good test–retest reliability (ICC = 0.75).
3. Patient Assessment of Constipation Quality of Life (PAC-QOL) (9): PAC-QOL, developed by Marquis et al., is utilized to evaluate the quality of life in patients with constipation. This instrument comprises four subscales: Concern, Physical Discomfort, Psychosocial Discomfort, and Satisfaction. The PAC-QOL has been validated and demonstrates strong internal consistency (Cronbach’s *α* > 0.80) and repeatability (ICC > 0.70).
4. Simplified Food Frequency Questionnaire (FFQ25) (10): The questionnaire, developed by Fudan University, is derived from the comprehensive Food Frequency Questionnaire (FFQ146) and typically includes about 25 food items or groups. It is designed to assess the frequency and quantity of food intake over a specified period, such as the past month or year. This semi-quantitative scale calculates a quantitative indicator by multiplying the frequency of food intake by the amount consumed per occasion, and then by corresponding coefficients. Additionally, the questionnaire was administered twice with a two-week interval, and the results showed no significant statistical difference between the two administrations, demonstrating high test–retest reliability with correlation coefficients ranging from 0.56 to 0.87.
5. Medical History: Chronicled prior medical conditions like hypertension, diabetes, and osteoarthritis of the knee.
6. Additional Factors: Included data on habits such as smoker, daily water consumption, salt intake, and weekly exercise routines.

### Determination of relevant outcomes

Evaluation of constipation: the PAC-SYM scale total score was used to classify constipation grades from normal, mild, moderate, severe, and very severe in descending order using the quintile spacing method.

Body Mass Index (BMI) was calculated using the formula: BMI = weight (kg) ÷ height (m)^2^. The classification of BMI was based on the World Health Organization (WHO) guidelines (11). According to these guidelines, BMI categories are defined as follows: underweight is a BMI less than 18.5 kg/m^2^, normal weight is a BMI between 18.5 and 24.9 kg/m^2^, overweight is a BMI between 25.0 and 29.9 kg/m^2^, and obesity is defined as a BMI of 30.0 kg/m^2^ or greater.

Smoker: Smoker was defined as having smoked equal to or more than 100 cigarettes in the lifetime (12).

Hypertension, diabetes, hyperlipidemia, hyperuricemia and osteoarthritis of the knee: were defined as having been diagnosed by a physician in the past.

### Statistical analysis

All calculations were rigorously adjusted to accurately reflect the adult population in Urumqi aged 20 years and older, as per the 2019 population census. The weighting scheme, developed using both census data and our sampling framework, was specifically designed to correct for any oversampling biases. For instance, lower weights were assigned to individuals of other nationality and female where oversampling occurred, and higher weights were applied to underrepresented groups. This approach ensured a balanced representation, compensating for potential biases due to oversampling, non-participation, and demographic discrepancies between our sample and the general population. Consequently, the age-standardized prevalence rates for conditions like constipation, derived from these adjustments, provide a more accurate reflection of the community’s health status.

Statistical significance for continuous variables was assessed using either Analysis of Variance (ANOVA) or Student’s t-test, depending on the distribution and characteristics of the data. For categorical variables, the χ^2^ (Chi-square) test was employed. Principal component analysis was applied to the data from the FFQ25 questionnaire to discern dietary patterns. Multiple linear regression analyses were conducted to identify covariates and explore the relationship between various risk factors and PAC-SYM scores. Using partial correlation to analyze the association between PAC-SYM scores and PAC-QOL scores.

To enhance the validity of our results, we implemented the Benjamini-Hochberg procedure to adjust *p*-values for multiple comparisons. This method effectively controls the false discovery rate, while adhering to the standard significance level of *α* = 0.05. Accordingly, adjusted *p*-values below 0.05 were interpreted as statistically significant. This rigorous approach ensures that our statistically significant findings are reflective of genuine associations, rather than artifacts of multiple testing. All statistical analyses were performed using a two-sided test approach, utilizing R software (version 4.3.0).

### Role of the funding source

The funder of this study are the major members in Xinjiang Medical University, who designed these studies in cooperation with other members of Xinjiang Medical University. The funder had a role in data collection, data analysis, data interpretation, and writing of the report. The funder cooperates with other members of Xinjiang Medical University to interpret the data. Funder authors collaborated with academic authors in the development of the manuscript.

## Results

### Participant constipation severity

Of the 1,386 participants surveyed, 64 (4.6%) exhibited mild constipation, 269 (19.4%) had moderate constipation, 281 (20.3%) suffered from severe constipation, and 257 (18.5%) were categorized with extremely severe constipation. Table 1 summarizes the distribution of adults with varying constipation severity in 2023, categorized by general characteristics and risk factors. The age-standardized prevalence rates for mild, moderate, severe, and very severe constipation were 4.7, 19.1, 19.3, 17.6%. Upon stratifying by gender, in the male cohort, the age-standardized prevalence rates for mild, moderate, severe, and extremely severe constipation were 6.8% (95%CI: 4.9 ~ 9.3), 25.9% (95%CI: 22.3 ~ 29.7), 28.5% (95%CI: 24.8 ~ 32.5), and 34.3% (95%CI: 30.4 ~ 38.4), respectively. For females, these rates were 8.8% (95%CI: 6.9 ~ 10.9), 35.9% (95%CI: 32.6 ~ 39.2), 34.6% (95%CI: 31.4 ~ 38.0), and 26.2% (95%CI: 23.2 ~ 29.3). Detailed age-standardized prevalence rates for constipation severity across different characteristics can be found in Supplementary Table S1. When stratified by ethnicity, in the Han population, the age-standardized prevalence rates for mild, moderate, severe, and extremely severe constipation were 7.7% (95%CI: 6.0 ~ 9.6), 34.1% (95%CI: 31.0 ~ 37.2), 29.8% (95%CI: 26.8 ~ 32.8), and 26.0% (95%CI: 23.2 ~ 28.9), respectively. In other ethnic groups, these rates were 8.3% (95%CI: 6.0 ~ 11.2), 28.3% (95%CI: 24.3 ~ 32.6), 38.4% (95%CI: 34.0 ~ 43.0), and 37.8% (95%CI: 33.4 ~ 42.4). Detailed rates for different characteristics are provided in Supplementary Tables S1, S2.

**Table 1:** Demographics and risk factors by constipation in Urumqi city of adults population in 2023.

	Normal	Mild	Moderate	Severe	Very severe
Participants	515 (37.2%)	64 (4.6%)	269 (19.4%)	281 (20.3%)	257 (18.5%)
Male	233 (45.2%)	24 (37.5%)	86 (32.0%)	94 (33.5%)	120 (46.7%)
Female	282 (54.8%)	40 (62.5%)	183 (68.0%)	187 (66.5%)	137 (53.3%)
Mean age (years)	42.3 (16.6)	41.8 (16.4)	38.3 (14.3)	37.1 (13.5)	37.2 (12.9)
Han nationality	365 (70.9%)	41 (64.1%)	195 (72.5%)	172 (61.2%)	148 (57.6%)
Other nationality	150 (29.1%)	23 (35.9%)	74 (27.5%)	109 (38.8%)	109 (42.4%)
Urban residents	448 (87.0%)	57 (89.1%)	223 (82.9%)	223 (79.4%)	161 (62.6%)
Rural residents	59 (11.5%)	6 (9.4%)	42 (15.6%)	53 (18.9%)	75 (29.2%)
Suburb residents	8 (1.6%)	1 (1.6%)	4 (1.5%)	5 (1.8%)	21 (8.2%)
**Education attainment**					
Primary school and lower	46 (8.9%)	7 (10.9%)	15 (5.6%)	30 (10.7%)	36 (14.0%)
Middle and high school	153 (29.7%)	11 (17.2%)	48 (17.8%)	44 (15.7%)	86 (33.5%)
College and higher	316 (61.4%)	46 (71.9%)	206 (76.6%)	207 (73.7%)	135 (52.5%)
**BMI (kg/m** ^**2**^ **)**					
Underweight	40 (7.8%)	12 (18.8%)	24 (8.9%)	33 (11.7%)	19 (7.4%)
Normal	315 (7.8%)	31 (48.4%)	166 (61.7%)	164 (58.4%)	173 (67.3%)
Overweight	130 (25.2%)	19 (29.7%)	63 (23.4%)	64 (22.8%)	49 (19.1%)
Obesity	30 (5.8%)	2 (3.1%)	16 (5.9%)	20 (7.1%)	16 (6.2%)
Nature of work					
Office work	157 (30.5%)	22 (34.4%)	113 (42.0%)	107 (38.1%)	65 (25.3%)
Manual labor	167 (32.4%)	24 (37.5%)	83 (30.9%)	91 (32.4%)	101 (39.3%)
Outdoor work	26 (5.0%)	3 (4.7%)	5 (1.9%)	16 (5.7%)	49 (19.1%)
Other	165 (32.0%)	15 (23.4%)	68 (25.3%)	67 (23.8%)	42 (16.3%)
Smoker	86 (16.1%)	10 (15.6%)	34 (12.6%)	34 (12.1%)	53 (20.6%)
Daily water intake ≥1,000 mL/day	293 (56.9%)	32 (50.0%)	132 (49.1%)	109 (38.8%)	65 (25.3%)
Daily salt consumption ≥6 g	440 (85.4%)	53 (82.8%)	234 (87.0%)	256 (91.1%)	240 (93.4%)
**Hours of physical activity per week**					
<1 h	243 (47.2%)	30 (46.9%)	165 (61.3%)	164 (58.4%)	108 (42.0%)
1 ~ 2.5 h	167 (32.4%)	20 (31.3%)	71 (26.4%)	92 (32.7%)	110 (42.8%)
≥2.5 h	105 (20.4%)	14 (21.9%)	33 (12.3%)	25 (8.9%)	39 (15.2%)
Sleep disorder	114 (22.1%)	16 (25.0%)	82 (30.5%)	94 (33.5%)	120 (46.7%)
Regularity in one’s daily life	331 (64.3%)	38 (59.4%)	142 (52.8%)	128 (45.6%)	110 (42.8%)
Hypertension	76 (14.8%)	10 (15.6%)	44 (16.4%)	39 (13.9%)	85 (33.1%)
Diabetes	32 (6.2%)	2 (3.1%)	18 (6.7%)	26 (9.3%)	39 (15.2%)
Hyperlipidemia	17 (3.3%)	7 (10.9%)	15 (5.6%)	19 (6.8%)	33 (12.8%)
Hyperuricemia	9 (1.7%)	1 (1.6%)	11 (4.1%)	8 (2.8%)	12 (4.7%)
Osteoarthritis of the knee	38 (7.4%)	5 (7.8%)	22 (8.2%)	55 (19.6%)	73 (28.4%)

Data are shown as number (%) or mean (SE). Smoker was defined as having smoked equal to or more than 100 cigarettes in the lifetime. According to the Chinese BMI index classification, BMI < 18.5 kg/m^2^ is underweight, 18.5 ≤ BMI < 24 kg/m^2^ is normal, 24 ≤ BMI < 28 kg/m^2^ is overweight, and ≥28 kg/m^2^ is obesity. Hypertension, diabetes, hyperlipidemia, hyperuricemia and osteoarthritis of the knee were defined as having been diagnosed by a physician in the past.

### Dietary patterns

The 25 dietary factors from the FFQ25 questionnaire were incorporated into a factor analysis model. The principal component method was used to extract common factors, with the maximum convergence iteration set at 25 times and coefficients with absolute values below 0.60 being suppressed. The results of the factor analysis correlation test were as follows: KMO value = 0.85, Bartlett’s test of sphericity (χ^2^ =8203.06, *p* < 0.0001). The 13 factors included in the analysis showed strong correlations and were suitable for factor analysis. Factors with an eigenvalue greater than 1 were retained, resulting in the extraction of six principal components. These were categorized into six dietary patterns: (1) Dietary pattern 1: Primarily consisting of white wine, yellow wine, sweets, snacks, cakes, and beer. (2) Dietary pattern 2: Dominated by red meat dishes (such as pork, beef, and mutton), poultry dishes (like chicken, duck, and goose), and chicken or duck eggs. (3) Dietary pattern 3: Mainly composed of coarse grains (including brown rice, millet, corn, barley, oats, adzuki beans, mung beans, etc.) and tubers (including sweet potatoes, potatoes, taro, yam, and konjac). (4) Dietary pattern 4: Predominantly featuring dark-colored vegetable dishes (such as bok choy, spinach, water spinach, tomatoes, green peppers, carrots, etc.) and light-colored vegetable dishes (like cabbage, radish, and cucumber). (5) Dietary pattern 5: Centered around seafood dishes (such as belt fish, pomfret, yellow croaker, and shrimp). (6) Dietary pattern 6: Mainly based on rice. For more details, refer to Supplementary Table S1 and Supplementary Figure S2.

### Multivariate linear regression analysis

The analysis revealed several factors positively correlated with PAC-SYM scores, including residing in rural areas compared to urban areas, outdoor work compared to office work, weekly exercise duration of 1–2.5 h compared to less than 1 h, sleep disorders, hypertension, knee osteoarthritis, and dietary patterns 1 and 5 (all *p* < 0.05). Factors negatively correlated with PAC-SYM scores included age, being overweight compared to having a normal BMI, daily water intake exceeding 1,000 mL, regular daily routines, and dietary patterns 2, 4, and 6 (all *p* < 0.05).For more details, refer to Table 2. Detailed findings stratified by gender and ethnicity are provided in Supplementary Table S4.

**Table 2:** Multiple linear regression analysis of PAC-SYM total scores of adults in Urumqi city.

	β	95%CI	*p*
Constants	9.43	4.63 ~ 14.24	0.0001
Women	−0.07	−1.15 ~ 1.00	0.8948
Age (10 year increments)	−0.97	−1.41 ~ −0.53	<0.0001
Other nationality	0.53	−0.48 ~ 1.55	0.3029
Rural resident	1.96	0.63 ~ 3.29	0.0039
Suburb residents	4.39	1.46–7.32	0.0034
**Education attainment**			
Primary school and lower	0.00	–	
Middle and high school	0.16	−1.61 ~ 0.94	0.8584
College and higher	−1.27	−3.08 ~ 0.54	0.1680
**BMI**			
Underweight	−0.93	−2.59 ~ 0.73	0.2718
Normal	0.00	–	
Overweight	−1.17	−2.32 ~ −0.03	0.0448
Obesity	−1.26	−3.28 ~ 0.75	0.2183
**Nature of work**			
Office work	0.00	–	
Manual labor	0.63	−0.54 ~ 1.80	0.2878
Outdoor work	2.65	0.57 ~ 4.73	0.0127
Other	−1.01	−2.27 ~ 0.24	0.11
Smoker	0.28	−1.13 ~ 1.69	0.6987
Daily water intake ≥1,000 mL/day	−2.45	−3.43 ~ −1.47	<0.0001
Daily salt consumption ≥6 g	1.43	−0.04 ~ 2.90	0.0559
**Hours of physical activity per week**			
<1 h	0.00	–	
1 ~ 2.5 h	1.35	0.28 ~ 2.43	0.0139
≥2.5 h	0.57	−0.83 ~ 1.97	0.4232
Sleep disorder	2.66	1.62 ~ 3.71	<0.0001
Regularity in one’s daily life	−2.19	−3.18 ~ −1.20	<0.0001
Hypertension	3.68	2.35 ~ 5.01	<0.0001
Diabetes	1.54	−0.23 ~ 3.32	0.0887
Hyperlipidemia	2.22	0.26 ~ 4.19	0.0263
Hyperuricemia	−1.20	−4.00 ~ 1.59	0.3984
Osteoarthritis of the knee	4.35	2.90 ~ 5.80	<0.0001
Dietary pattern 1	1.16	0.66 ~ 1.65	<0.0001
Dietary pattern 2	−0.70	−1.17 ~ −0.22	0.0040
Dietary pattern 3	−0.32	−0.80 ~ 0.15	0.1857
Dietary pattern 4	−0.54	−1.03 ~ −0.04	0.0354
Dietary pattern 5	1.13	0.64 ~ 1.61	<0.0001
Dietary pattern 6	−0.99	−0.47 ~ −0.51	0.0001

Multiple linear regression analysis includes all covariates listed in the table. β of 0 indicate reference values. Hypertension, diabetes, hyperlipidemia, hyperuricemia and osteoarthritis of the knee were defined as having been diagnosed by a physician in the past time. Smoker was defined as having smoked equal to or more than 100 cigarettes in the lifetime. According to the Chinese BMI index classification, BMI < 18.5 kg/m^2^ is malnutrition, 18.5 ≤ BMI < 24 kg/m^2^ is normal, 24 ≤ BMI < 28 kg/m^2^ is overweight, and ≥28 kg/m^2^ is obesity.

### Association between constipation severity and PAC-QOL metrics

**Figure 1:**
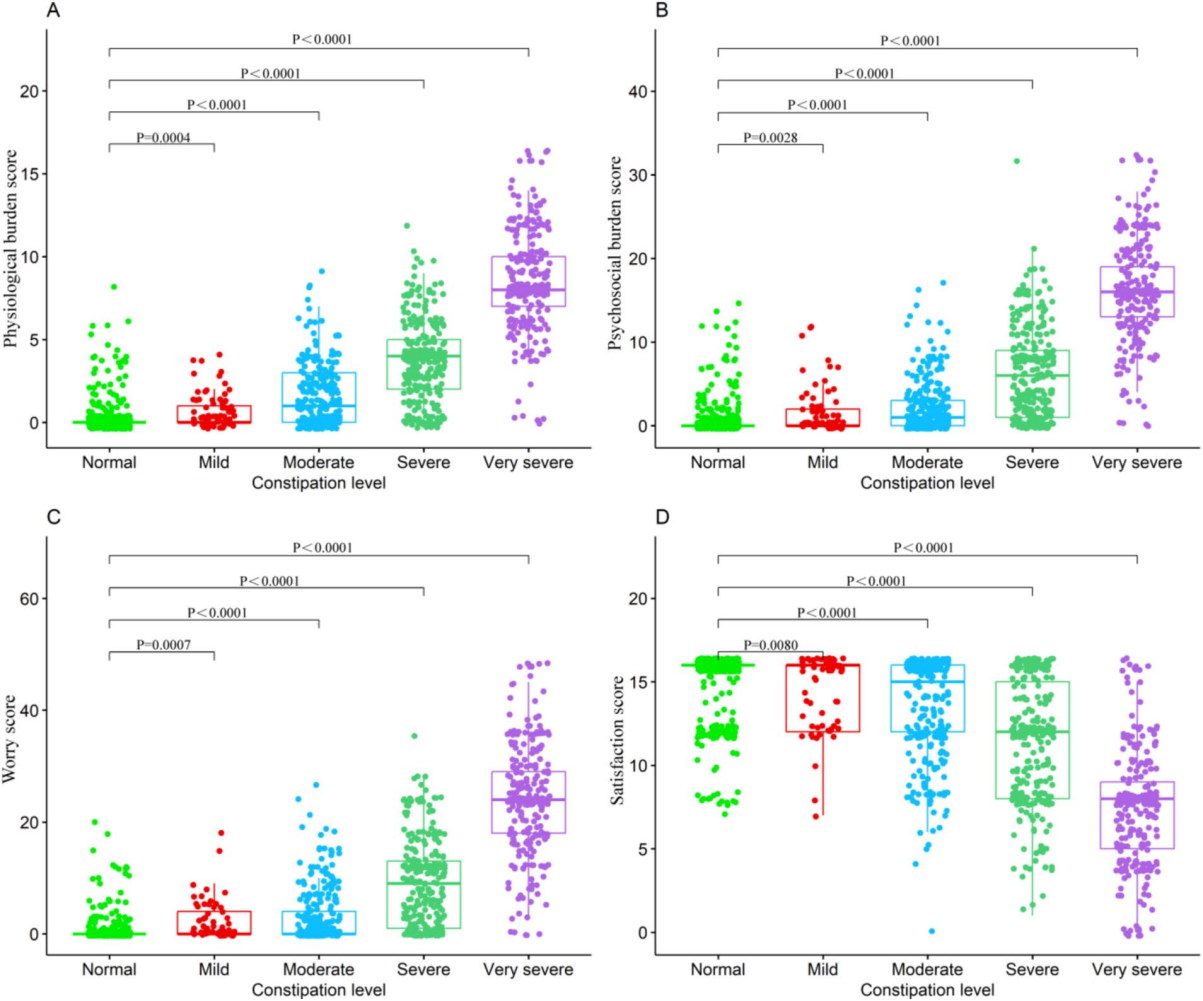
Association between consipation level and PAC-QOL metrics **(A)** Physicligical burden score. **(B)** Psychosocial burden score. **(C)** Worry score. **(D)** Satisfaction score.

A pronounced correlation was observed between the severity of constipation and various dimensions of the PAC-QOL scale. Specifically, as constipation severity escalated, there was a discernible increase in scores related to physiological strain, psychosocial impact, and concerns. Conversely, satisfaction scores exhibited a decline (all *p* < 0.05), further details can be found in Figure 1. Upon executing a partial correlation analysis with multivariate adjustments, the PAC-SYM scores exhibited a positive association with PAC-QOL metrics for physiological strain (*r* = 0.87, *p* < 0.05), psychosocial impact (*r* = 0.86, *p* < 0.05), and concerns (*r* = 0.86, *p* < 0.05). A negative association was evident with satisfaction (*r* = −0.73, *p* < 0.05), detailed results are shown in Table 3.

**Table 3:** Correlation analysis of PAC-SYM score and PAC-QOL score by gender and nationality.

	Man		Woman		Han nationality		Other nationality		Total	
	*r*	*p*	*r*	*p*	*r*	*p*	*r*	*p*	*r*	*p*
Physiological burden score	0.89	<0.0001	0.85	<0.0001	0.88	<0.0001	0.85	<0.0001	0.87	<0.0001
Psychosocial burden score	0.90	<0.0001	0.81	<0.0001	0.87	<0.0001	0.84	<0.0001	0.86	<0.0001
Worry score	0.88	<0.0001	0.83	<0.0001	0.87	<0.0001	0.83	<0.0001	0.86	<0.0001
Satisfaction score	−0.78	<0.0001	−0.69	<0.0001	−0.74	<0.0001	−0.70	<0.0001	−0.73	<0.0001

## Discussion

Urumqi, the capital of Xinjiang Uyghur Autonomous Region, is a multicultural hub with diverse ethnic groups such as the Han, Uyghur, Kazakh, Hui, Mongolian, and more. This rich cultural diversity offers a unique opportunity for epidemiological studies on constipation. Different ethnicities and cultures may have distinct lifestyle practices, dietary habits, and health beliefs influencing the prevalence of constipation.

The prevalence of constipation observed in our study in Urumqi showed variation when compared to other regions. For instance, studies in North America reported a constipation prevalence of 16.6% (13), and in Europe and Oceania, it was 17.1% (14). However, our findings indicated higher prevalence rates, particularly among certain subgroups. These discrepancies may be attributed to several factors, including methodological differences, such as variations in diagnostic criteria (e.g., Rome III vs. Rome IV) and the use of different assessment tools for symptom severity (15). Additionally, population characteristics specific to Urumqi may contribute to the higher prevalence. Urumqi’s unique geographic and climatic conditions, such as its dry, cold environment, could influence gastrointestinal function, potentially exacerbating symptoms of constipation. Furthermore, cultural and dietary practices in this multicultural region might differ from those in Western countries, where diets typically include higher fiber content, which has been shown to mitigate constipation risks (16). The ethnic diversity in Urumqi also adds complexity. Our findings revealed that Han Chinese participants had lower rates of constipation compared to other ethnic groups, which may reflect differences in dietary habits and lifestyle practices. For example, traditional diets among Uyghur and Kazakh populations often emphasize meats and fewer fiber-rich foods, potentially contributing to the increased constipation rates observed in these groups (17).

In our study, we explored various factors contributing to constipation. We highlighted the complex interplay between lifestyle, dietary habits, socio-demographic factors, health conditions, sleep, and ethnicity in urban and rural populations. Sedentary lifestyles, typical of urban living, have been linked with a higher risk of constipation (18). Diets with low fiber intake and high processed food consumption are significant contributors to this condition (19). Our findings highlight the importance of physical activity for gut motility. Individuals engaging in less than 1 h of exercise per week had higher PAC-SYM scores, indicating more severe constipation. Dietary patterns reliant on processed foods and low fiber intake correlated with increased constipation severity (20). We observed a higher likelihood of constipation among rural and suburban residents compared to urban ones. These disparities might be due to variations in dietary preferences, healthcare facility access, and different lifestyle factors in various geographic settings (14). Our study also found a positive correlation between health conditions like hypertension, hyperlipidemia, and osteoarthritis and the PAC-SYM score. Chronic medical conditions can affect bowel habits through medication side effects, reduced physical activity, and dietary restrictions (21). Sleep disturbances and constipation severity were positively correlated in our study. Poor sleep quality affects gut motility and disrupts the gut-brain axis, leading to constipation (22). Stress and psychological factors also impact bowel movements, emphasizing the connection between mental and gut health (23). Our study’s setting in Urumqi allowed us to explore ethnicity’s influence on constipation patterns. Han Chinese individuals had distinct constipation patterns compared to other ethnic groups, possibly due to genetic predispositions and the interplay of cultural and dietary factors. This observation underscores the need for further research to understand the mechanisms driving constipation prevalence variations among different ethnic groups.

A significant finding from our study is constipation’s profound impact on quality of life, as shown by the correlation with PAC-QOL scores. The PAC-QOL scale, a validated tool for assessing constipated individuals’ quality of life, has highlighted constipation’s multifaceted burden (24). Higher constipation severity correlated with increased physiological and psychosocial burdens, emphasizing the need to address constipation’s psychological and social implications alongside its physical aspects (25). Previous studies have also highlighted the psychological distress and reduced social interactions experienced by individuals with severe constipation (26). The negative correlation with satisfaction further underscores the diminished quality of life in these individuals.

Constipation management has shifted over the years from symptom alleviation to addressing root causes. Dietary modifications, especially increased fiber intake, remain the cornerstone of constipation management. A meta-analysis highlighted the efficacy of specific foods, such as kiwifruit and rye bread, in improving stool frequency and gut transit time in adults with chronic constipation, suggesting dietary modifications as effective intervention (16). In culturally unique areas like Urumqi, public health strategies must consider traditional dietary patterns that might be contributing to constipation. Populations such as the Uyghur and Kazakh, whose diets traditionally include lower fiber content, would benefit from culturally tailored dietary interventions. Programs promoting the inclusion of fiber-rich foods and improved hydration can help address the high prevalence of constipation in these communities (27). These interventions should be designed in collaboration with local communities to ensure cultural relevance, as has been demonstrated in studies involving transcultural dietary interventions in immigrant populations (28). Pharmacological interventions, including osmotic laxatives like lactulose and polyethylene glycol, have also been proven effective. However, integrating these medications into culturally sensitive treatment protocols is essential. Newer agents, such as lubiprostone and linaclotide, have shown promise, but their long-term impact, particularly in populations with limited healthcare access, requires further study. For Urumqi, where access to healthcare varies, expanding education on these treatment options could improve patient outcomes. Behavioral therapies, such as biofeedback, have been effective for patients with dyssynergic defecation. Integrating these therapies into local health systems, particularly in rural areas, can help address constipation where lifestyle factors such as physical inactivity and stress play a significant role. Additionally, alternative therapies like acupuncture, which align with local cultural practices, could be incorporated into a holistic approach to managing constipation in Urumqi (29).

This study has several limitations that should be considered when interpreting the findings. First, the cross-sectional design limits our ability to draw causal inferences between the identified risk factors and constipation, and further longitudinal research is needed to confirm these associations. Second, as the data were collected through self-reported questionnaires, recall bias may have influenced the accuracy of participants’ responses regarding their dietary habits, physical activity, and constipation symptoms. Third, some subgroups, such as suburban residents, had small sample sizes, which reduced the statistical power and generalizability of the results for these groups. While we retained these categories to preserve the representativeness of Urumqi’s diverse population, the findings related to these smaller groups should be interpreted with caution, and future studies with larger sample sizes are warranted. Finally, although our sample was stratified to reflect the age, gender, and ethnic distribution of Urumqi’s population, generalizability to other regions or populations may be limited due to differences in lifestyle, healthcare access, and dietary practices. Additional research in more diverse populations is needed to validate these results and assess their broader applicability.

In conclusion, while significant progress has been made in managing constipation, a holistic approach incorporating dietary, pharmacological, and behavioral therapies is crucial. For regions like Urumqi, culturally tailored interventions that account for local dietary and lifestyle habits can greatly enhance the effectiveness of public health strategies.

## Conclusion

In this study, we found that constipation in Urumqi was significantly associated with lifestyle, dietary, and health-related factors. Higher prevalence was observed in rural and suburban residents, those with sleep disorders, and individuals with specific dietary patterns, while increased water intake and certain diets were linked to lower constipation severity. These findings suggest that public health interventions should focus on promoting culturally tailored dietary and lifestyle changes to manage constipation. Healthcare providers should incorporate these factors into personalized treatment strategies. Future research should explore these associations further through longitudinal studies and evaluate the effectiveness of targeted interventions.

## Data Availability

The raw data supporting the conclusions of this article will be made available by the authors, without undue reservation.
