# Supplementary material for: Prevalence, risk factors, impact and management of constipation among adults in Urumqi, China: a cross-sectional survey
Source: Front Nutr. 2024 Nov 1;11:1451527. doi: 10.3389/fnut.2024.1451527 (PMC11563834; doi:10.3389/fnut.2024.1451527)
Supplement: Supplementary file 1 [file Data_Sheet_1.pdf]

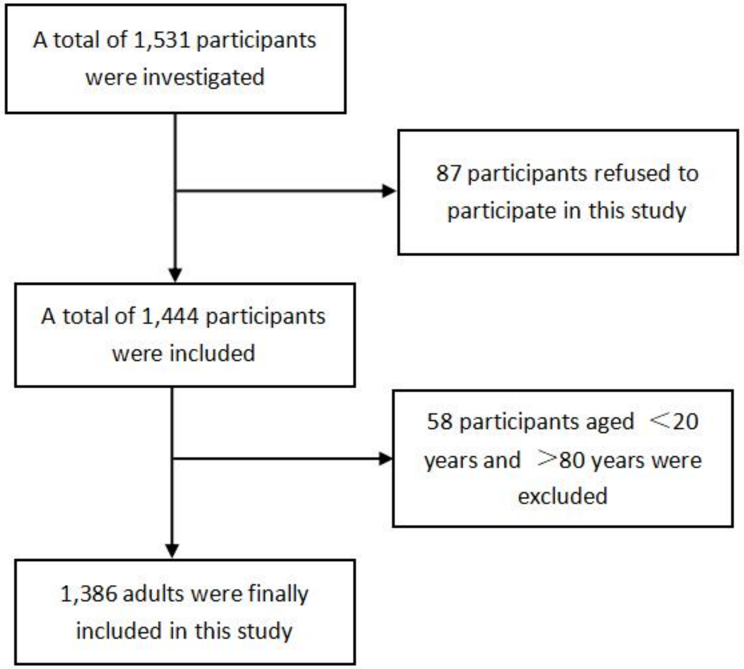

Figure S1. Flowchart of this study

Table S1. Age-specific and age-standardized prevalence of constipation among adults in the Urumqi city by gender

	Man					Woman					Total				
Age(years)	Normal	Mild	Moderate	Severe	Very severe	Normal	Mild	Moderate	Severe	Very severe	Normal	Mild	Moderate	Severe	Very severe
20~29	37.6%(30.5-45.1)	3.9%(1.6-7.8)	16.6%(11.5-22.8)	17.1%(11.9-23.4)	24.9%(18.7-31.8)	33.9%(28.4-39.8)	4.2%(2.2-7.3)	22.6%(17.9-27.9)	25.1%(20.1-30.6)	14.1%(10.3-18.7)	35.3%(31.0-39.9)	4.1%(2.5-6.3)	20.3%(16.7-24.2)	22.0%(18.3-26.0)	18.3%(14.9-22.1)

30~39	35.2%(26.9-44.1)	6.3%(2.7-11.9)	14.1%(8.6-21.3)	18.0%(11.7-25.7)	26.6%(19.1-35.1)	24.3%(18.8-30.5)	4.1%(1.9-7.6)	23.4%(18-29.6)	25.7%(20.1-31.9)	22.5%(17.2-28.6)	28.3%(23.6-33.3)	4.9%(2.9-7.7)	20.0%(15.9-24.6)	22.9%(18.6-27.6)	24.0%(19.6-28.8)
40~49	31.5%(21.1-43.4)	0.0%(0.0-4.9)	19.2%(10.9-30.1)	23.3%(14.2-34.6)	26.0%(16.5-37.6)	29.9%(22.1-38.7)	4.7%(1.8-10.0)	22.0%(15.2-30.3)	23.6%(16.5-32.0)	19.7%(13.2-27.7)	30.5%(24.2-37.4)	3.0%(1.1-6.4)	21.0%(15.6-27.3)	23.5%(17.8-30)	22.0%(16.5-28.4)
50~59	49.3%(37.4-61.3)	5.5%(1.5-13.4)	15.1%(7.8-25.4)	16.4%(8.8-27.0)	13.7%(6.8-23.8)	44.0%(34.1-54.3)	4.0%(1.1-9.9)	23%(15.2-32.5)	15.0%(8.6-23.5)	14.0%(7.9-22.4)	46.2%(38.6-54.0)	4.6%(2-8.9)	19.7%(14.0-26.4)	15.6%(10.5-21.9)	13.9%(9.1-19.9)
≥60	59.5%(49.6-69.4)	4.9%(1.6-11.1)	12.7%(7-20.8)	10.8%(5.5-18.5)	11.8%(6.2-19.6)	51.5%(41.2-61.8)	9.3%(4.3-16.9)	16.5%(9.7-25.4)	14.4%(8.1-23.0)	8.2%(3.6-15.6)	55.8%(48.6-62.8)	7.0%(3.9-11.5)	14.6%(10.0-20.3)	12.6%(8.3-18.0)	10.1%(6.2-15.1)
P value for trend	0.0002	0.8698	0.5256	0.2559	0.0024	0.0004	0.1279	0.3283	0.0067	0.2089	＜0.0001	0.2203	0.1701	0.0028	0.0043
Nationality															
Han nationality	76.9%(72.3-81.1)	6.5%(4.3-9.5)	27.1%(22.7-31.9)	25.4%(21.1-30.1)	30.7%(26.1-35.6)	63.9%(59.7-68.0)	8.7%(6.4-11.4)	38.9%(34.8-43.1)	32.8%(28.8-36.9)	22.4%(19.0-26.2)	69.2%(66.1-72.2)	7.7%(6.0-9.6)	34.1%(31.0-37.2)	29.8%(26.8-32.8)	26.0%(23.2-28.9)
Other nationality	55.6%(48.0-63.1)	7.7%(4.3-12.7)	23.4%(17.3-30.3)	38.0%(30.9-45.6)	41.9%(34.5-49.5)	52.8%(46.9-58.7)	9.1%(6.0-13.0)	31.2%(25.9-36.9)	37.9%(32.3-43.8)	35.6%(30.0-41.4)	53.8%(49.1-58.4)	8.3%(6.0-11.2)	28.3%(24.3-32.6)	38.4%(34.0-43.0)	37.8%(33.4-42.4)
P value	＜0.0001	0.7240	0.4021	0.0032	0.0128	0.0025	0.9332	0.0351	0.1563	0.0001	＜0.0001	0.7397	0.0353	0.0015	＜0.0001

for difference															
Urbanisation															
Urban	76.5%(72.2-80.4)	7.3%(5.0-10.1)	30.6%(26.3-35.2)	25.2%(21.2-29.6)	27.1%(22.9-31.5)	67.6%(63.9-71.1)	9.7%(7.6-12.2)	33.0%(29.5-36.7)	35.6%(32.0-39.4)	20.7%(17.7-24)	71.2%(68.4-73.8)	8.6%(7.0-10.4)	32.2%(29.4-35)	31.7%(28.9-34.5)	23.1%(20.6-25.7)
Rural	46.7%(36.9-56.6)	5.0%(1.7-11.0)	8.1%(3.7-15)	44.2%(34.5-54.2)	62.7%(52.8-71.9)	33.3%(25.2-42.1)	4.4%(1.6-9.5)	47.6%(38.7-56.5)	33.8%(25.8-42.7)	47.6%(38.7-56.5)	38.6%(32.3-45.1)	4.3%(2.1-7.8)	31.8%(25.9-38.2)	39.0%(32.7-45.6)	52.9%(46.3-59.4)
Suburb	31.0%(11.3-57.5)	11.1%(1.3-35.6)	11.1%(1.3-35.6)	21.4%(5.7-47.6)	92.1%(68.5-99.5)	26.7%(10.3-49.6)	0.0%(0.0-15.4)	37.5%(18.1-60.4)	6.7%(0.5-25.9)	62.5%(39.6-81.9)	31.2%(17.4-48.0)	11.1%(3.3-25.3)	33.3%(19.1-50.2)	11.4%(3.5-25.7)	79.6%(63.6-90.8)
P value for difference	< 0.0001	0.8273	< 0.0001	0.0090	< 0.0001	< 0.0001	0.0147	0.0097	0.0388	< 0.0001	< 0.0001	0.2160	0.9850	0.9406	< 0.0001
Education attainment															
Primary	55.7%(42.8-68	6.6%(1.9-15.6	6.2%(1.7-15.1)	42.1%(30.0-55	56.1%(43.2-68	54.8%(42.3-66	7.3%(2.4.0-16.	23.2%(13.9-34	39.7%(28.1-52	41.8%(30.0-54	54.0%(45.1-62	7.0%(3.3-12.7	16.2%(10.4-23	39.4%(31.1-48	50.2%(41.4-58

school and lower	.0)	)		.0)	.4)	.8)	2)	.9)	.2)	.3)	.6)	)	.5)	.2)	.9)
Middle and high school	59.2%(51.1-67)	6.2%(3.0-11.1)	20%(14.1-27.1)	17.4%(11.8-24.2)	63.9%(55.9-71.3)	71.5%(64.4-77.9)	5.5%(2.7-9.9)	23.7%(17.7-30.5)	26.5%(20.2-33.4)	39.5%(32.4-47)	66.4%(61.2-71.4)	5.7%(3.5-8.7)	22.4%(18.1-27.2)	22.1%(17.8-26.9)	50.0%(44.6-55.4)
College and higher	77.7%(72.8-82.0)	8.5%(5.7-12.0)	30.5%(25.6-35.7)	29.4%(24.5-34.6)	20.6%(16.4-25.4)	49.1%(45.0-53.3)	13.7%(11.0-16.8)	41.0%(37.0-45.2)	36.8%(32.8-40.9)	26%(22.4-29.7)	61.1%(57.8-64.2)	11.1%(9.1-13.3)	36.4%(33.3-39.6)	34.3%(31.3-37.5)	23.8%(21-26.7)
P value for trend	＜0.0001	0.4047	＜0.0001	0.6625	＜0.0001	0.0006	0.0049	＜0.0001	0.3317	0.0001	0.7042	0.0085	＜0.0001	0.2631	＜0.0001
BMI															
Malnutrition	55.6%(32.6-76.9)	25.0%(9.0-48.4)	11.1%(1.7-32.4)	36.1%(16.6-59.7)	38.9%(18.7-62.3)	67.2%(57.4-76.0)	9.9%(5.0-17.2)	24.9%(17.0-34.1)	37.7%(28.5-47.6)	27.1%(18.9-36.5)	67.1%(58.3-75.2)	14.3%(8.8-21.6)	21%(14.3-29.1)	34.4%(26.3-43.3)	29.7%(22.0-38.5)
Normal	72.5%(67.4-77.2)	5.8%(3.6-8.9)	23.9%(19.4-28.8)	25.4%(20.8-30.4)	39.1%(33.9-44.5)	60.9%(56.5-65.2)	6.6%(4.6-9.1)	37.5%(33.3-41.9)	34.4%(30.3-38.7)	27.3%(23.4-31.4)	65.0%(61.7-68.2)	6.2%(4.7-8.1)	32.9%(29.7-36.1)	31.0%(27.9-34.3)	31.5%(28.4-34.8)
Overweight	76.6%(69.2-82.9)	6.1%(2.9-11.1)	29.6%(22.6-37.3)	31%(23.9-38.9)	23.4%(17.0-30.8)	56.1%(48.2-63.8)	12.1%(7.6-18.1)	34.6%(27.4-42.4)	35.9%(28.6-43.6)	27.9%(21.3-35.4)	66.0%(60.6-71.2)	9.6%(6.6-13.3)	32.4%(27.4-37.8)	33%(28.0-38.4)	25.6%(20.9-30.7)
Obesity	55.1%(38.3-71	7.5%(1.5-20.7	30.9%(17.1-47	41.4%(25.9-58	31.7%(17.8-48	76.1%(61.1-87	0.0%(0.0-7.9)	32.2%(19.1-47	36.0%(22.3-51	22.3%(11.3-37	65.8%(54.6-75	3.6%(0.7-10.1	30.0%(20.4-40	39.9%(29.4-51	27.4%(18.3-38

	)	)	.7)	.3)	.6)	.5)		.8)	.7)	.2)	.8)	)	.9)	.2)	.3)
P value for trend	0.7690	0.2898	0.0485	0.0822	0.0086	0.8394	0.7242	0.4339	0.9356	0.7521	0.9901	0.1980	0.2582	0.3180	0.1505
Nature of work															
Office work	68.7%(61.6-75.1)	7.7%(4.3-12.4)	37.4%(30.6-44.6)	26.8%(20.7-33.6)	26.1%(20.1-32.9)	54.2%(48.0-60.2)	11.1%(7.6-15.4)	40.9%(35.0-47.1)	41.2%(35.3-47.4)	19.2%(14.7-24.5)	61.2%(56.6-65.7)	9.4%(6.9-12.5)	39.1%(34.6-43.7)	35.0%(30.7-39.6)	21.9%(18.2-25.9)
Manual labor	68.4%(61-75.2)	7.2%(3.8-12.0)	15.7%(10.7-22)	32.6%(25.7-40.0)	42.8%(35.4-50.4)	60.9%(55.0-66.6)	9.1%(6.1-13.0)	34.1%(28.7-39.9)	31.9%(26.5-37.6)	30.7%(25.4-36.3)	63.4%(58.9-67.8)	8.3%(6-11.2)	25.8%(21.9-30.1)	32.5%(28.3-37)	36.5%(32.1-41.1)
Outdoor work	48.9%(35.4-62.5)	10.3%(3.8-21.3)	4.4%(0.8-13.5)	26.8%(15.9-40.2)	76.2%(63.1-86.5)	33.7%(19.8-49.9)	2.0%(0.0-11.9)	10.3%(3.1-23.6)	48.4%(32.7-64.3)	72.4%(56.4-85.0)	43.2%(33.3-53.6)	8.2%(3.7-15.5)	7.2%(3.0-14.2)	27.8%(19.2-37.7)	80.3%(71.1-87.6)
Other	89.3%(82.6-94.0)	5.7%(2.4-11.2)	26.7%(19.3-35.2)	29.3%(21.6-38.0)	15.7%(9.9-23.1)	70.5%(64.1-76.3)	6.2%(3.4-10.1)	33.4%(27.3-40.0)	35.7%(29.5-42.3)	20.9%(15.8-26.7)	76.4%(71.7-80.8)	5.9%(3.7-8.9)	32.2%(27.4-37.3)	32.6%(27.8-37.8)	19.5%(15.5-24.0)
P value for difference	0.0017	0.6475	0.0084	0.7711	0.5307	0.0017	0.0318	0.0399	0.4598	0.3657	0.0001	0.0715	0.0107	0.3906	0.5747
Somke															

r															
Yes	72.5%(65.5-78.8)	7.2%(3.9-11.9)	29%(22.6-36.1)	23%(17.2-29.8)	35%(28.1-42.3)	36.7%(19.7-56.6)	16.7%(5.5-35.1)	9.8%(1.9-26.6)	29.8%(14.4-49.6)	73.7%(54.1-88.2)	68.8%(62.2-75.0)	7.2%(4.1-11.5)	26.9%(21.1-33.4)	25%(19.3-31.4)	38.8%(32.2-45.6)
No	70.6%(65.7-75.2)	6.7%(4.4-9.7)	24.0%(19.7-28.6)	30.7%(26.1-35.7)	34.7%(29.9-39.8)	61.9%(58.5-65.3)	8.7%(6.8-10.9)	36.7%(33.3-40.1)	34.6%(31.3-38.0)	24.8%(21.8-27.9)	64.5%(61.7-67.3)	8.0%(6.5-9.7)	32.8%(30.1-35.5)	33.5%(30.8-36.3)	27.9%(25.3-30.5)
P value for difference	0.7044	0.9661	0.2389	0.0703	1.0000	0.0111	0.2514	0.0056	0.7395	＜0.0001	0.2539	0.7727	0.1073	0.0180	0.0018
Daily water intake															
＜1000mL	58.6%(52.6-64.3)	6.9%(4.2-10.5)	19.8%(15.4-24.9)	31.7%(26.4-37.5)	49.6%(43.7-55.6)	50.2%(45.6-54.8)	7.5%(5.3-10.3)	35.8%(31.5-40.4)	37.0%(32.6-41.5)	36.2%(31.8-40.7)	54.0%(50.4-57.6)	7.2%(5.5-9.3)	29.5%(26.3-32.9)	34.8%(31.4-38.3)	41.1%(37.5-44.7)
⩾1000mL	84.9%(80.1-89.0)	6.7%(4.0-10.4)	32.4%(26.8-38.3)	25.2%(20.2-30.8)	17.4%(13.1-22.5)	74.9%(70.1-79.3)	10.1%(7.2-13.7)	36.3%(31.3-41.5)	31.2%(26.5-36.3)	14.1%(10.7-18.2)	79.1%(75.7-82.2)	8.6%(6.6-11.1)	34.6%(30.9-38.4)	28.8%(25.3-32.5)	15.5%(12.8-18.6)
P value for difference	＜0.0001	1.0000	0.0010	0.1082	＜0.0001	＜0.0001	0.2271	0.9438	0.0964	＜0.0001	＜0.0001	0.3793	0.0505	0.0195	＜0.0001

Daily salt consumption															
＜6g	95.6%(86.5-99.3)	12.1%(4.9-23.6)	23.2%(13.0-36.4)	29.4%(18.0-43.1)	6.3%(1.6-16.2)	71.1%(61.6-79.5)	9.5%(4.7-16.7)	41.4%(32-51.4)	24.1%(16.3-33.3)	20.6%(13.4-29.5)	76.0%(68.6-82.3)	10.7%(6.4-16.5)	38.1%(30.6-46.0)	25.6%(19.1-33.1)	16.2%(10.9-22.8)
⩾6g	69.5%(65.3-73.5)	5.9%(4.0-8.3)	25.6%(21.9-29.7)	28.5%(24.6-32.6)	37.1%(32.9-41.5)	60.3%(56.6-63.9)	8.7%(6.8-11.0)	34.0%(30.6-37.6)	36.1%(32.6-39.7)	27.5%(24.2-30.9)	64.4%(61.7-67.1)	7.3%(5.9-8.9)	30.5%(27.9-33.2)	33.0%(30.4-35.7)	31.4%(28.8-34.1)
P value for difference	0.0001	0.1345	0.8145	1.0000	＜0.0001	0.0416	0.9439	0.1653	0.0194	0.1658	0.0048	0.1758	0.0599	0.0717	0.0001
Hours of physical activity per week															
＜1h	66.2%(59.6-72.2)	7.5%(4.5-11.7)	34.3%(28.2-40.9)	33.8%(27.7-40.3)	24.9%(19.4-31.0)	61.5%(57.0-65.9)	7.8%(5.6-10.6)	37.9%(33.6-42.4)	36.2%(31.9-40.7)	23.2%(19.5-27.3)	62.6%(58.9-66.2)	7.3%(5.5-9.5)	37.7%(34.1-41.4)	35.4%(31.9-39.0)	23.7%(20.6-27.0)
1~2.5h	65.4%(	4.8%(2	18.1%(	32.1%(	46.3%(	57.5%(	9.6%(6.	31.5%(	34.9%(	33.3%(	61.9%(	7.3%(5	24.8%(	33.6%(	39.1%(

	58.5-71.8)	.4-8.7)	13.2-24.0)	25.8-38.8)	39.4-53.2)	51.1-63.7)	2-13.9)	25.7-37.6)	29.0-41.2)	27.4-39.5)	57.3-66.4)	.1-10.0)	20.9-29.0)	29.3-38.1)	34.6-43.7)
≥2.5h	88.1%(80.7-93.3)	9.1%(4.6-15.9)	23.9%(16.5-32.7)	10.4%(5.5-17.5)	35.1%(26.5-44.6)	73.7%(63.9-82.0)	13.3%(7.3-21.5)	25.7%(17.5-35.4)	28.9%(20.3-38.9)	25.1%(17.0-34.8)	81.4%(75.5-86.3)	11.1%(7.2-16.1)	25.3%(19.7-31.7)	18.7%(13.7-24.5)	30.1%(24.1-36.7)
P value for trend	0.0002	0.8088	0.0062	＜0.0001	0.0058	0.1817	0.0842	0.0083	0.2107	0.1084	＜0.0001	0.1342	＜0.0001	＜0.0001	0.0004
Sleep disorder															
Yes	52.7%(44.7-60.6)	4.4%(1.8-8.8)	25.4%(18.9-32.9)	28.7%(21.8-36.3)	55.5%(47.5-63.3)	43.4%(37.4-49.6)	7.2%(4.4-11.0)	38.8%(32.9-44.9)	38.9%(33.0-45.0)	38.4%(32.5-44.5)	46.7%(41.9-51.6)	6.2%(4.1-8.9)	33.9%(29.4-38.6)	35.0%(30.4-39.7)	44.9%(40.1-49.8)
No	78.4%(74.0-82.3)	7.7%(5.2-10.7)	26.7%(22.4-31.4)	28.6%(24.2-33.3)	25.3%(21.1-29.9)	69.8%(65.8-73.6)	8.9%(6.7-11.6)	34.2%(30.3-38.2)	33.3%(29.4-37.3)	20.5%(17.3-24.1)	73.8%(70.9-76.6)	8.3%(6.6-10.2)	31.2%(28.2-34.2)	31.1%(28.2-34.2)	22.3%(19.7-25.1)
P value for difference	＜0.0001	0.2268	0.8319	1.0000	＜0.0001	＜0.0001	0.5008	0.2255	0.1359	＜0.0001	＜0.0001	0.2197	0.3397	0.1797	＜0.0001
Regularity in one's															

daily life															
Yes	75.5%(70.5-80.1)	6.1%(3.8-9.3)	29.5%(24.6-34.7)	23.7%(19.2-28.7)	31.8%(26.8-37.1)	71.2%(66.7-75.5)	9.8%(7.2-13.1)	34.0%(29.5-38.7)	32.4%(27.9-37.1)	19.2%(15.6-23.3)	73.3%(69.9-76.4)	8.1%(6.2-10.3)	32%(28.7-35.5)	28.9%(25.6-32.2)	24.5%(21.4-27.7)
No	60.7%(54.0-67.0)	7.2%(4.2-11.3)	17.9%(13.2-23.5)	38%(31.8-44.6)	42.9%(36.4-49.5)	47.4%(42.5-52.4)	5.3%(3.3-8.0)	35%(30.3-39.8)	41%(36.2-46.0)	38%(33.2-42.9)	52.7%(48.7-56.6)	6.3%(4.5-8.4)	28.3%(24.9-32.0)	39.2%(35.4-43.1)	40.2%(36.3-44.1)
P value for difference	0.0003	0.7502	0.0026	0.0004	0.0097	＜0.0001	0.0203	0.8207	0.0122	＜0.0001	＜0.0001	0.2322	0.1529	0.0001	＜0.0001
Hypertension															
Yes	42.4%(34.0-51.0)	3.9%(1.4-8.6)	29%(21.6-37.3)	24.3%(17.5-32.3)	67.1%(58.6-74.8)	44.4%(35.1-54.0)	6.1%(2.5-12.1)	26.2%(18.5-35.3)	33.5%(24.9-42.9)	56.5%(46.9-65.7)	43.0%(36.9-49.4)	5.3%(2.9-8.8)	27.2%(21.9-33.2)	27%(21.7-32.9)	64.1%(57.9-70.0)
No	81%(76.9-84.7)	8.0%(5.6-11.1)	22.2%(18.3-26.5)	29.6%(25.3-34.3)	25.8%(21.7-30.3)	66.3%(62.7-69.7)	8.2%(6.3-10.5)	35.6%(32.1-39.3)	35.4%(31.9-39.1)	21.1%(18.2-24.3)	71.5%(68.7-74.1)	8.3%(6.8-10.1)	30.8%(28.1-33.6)	33.1%(30.3-35.9)	23%(20.6-25.5)
P value for difference	＜0.0001	0.1453	0.1333	0.2756	＜0.0001	＜0.0001	0.5483	0.0627	0.7592	＜0.0001	＜0.0001	0.1268	0.2973	0.0733	＜0.0001

Diabetes															
Yes	46.8%(34.5-59.4)	2.8%(0.3-10.1)	17.3%(9.2-28.5)	33.3%(22.3-45.9)	66.5%(53.9-77.5)	29.9%(17.8-44.5)	2.4%(0.1-11.3)	36.1%(23.0-50.9)	46.8%(32.5-61.4)	51.5%(37.0-65.9)	38.7%(29.9-48.2)	2.7%(0.6-7.5)	24.9%(17.4-33.8)	39.2%(30.3-48.7)	61.1%(51.6-70.0)
No	73.7%(69.5-77.5)	7.6%(5.4-10.3)	26.7%(22.9-30.9)	27.9%(24-32.1)	30.7%(26.7-35)	64.0%(60.5-67.4)	9.3%(7.3-11.5)	35.2%(31.9-38.7)	34.0%(30.6-37.4)	24.2%(21.2-27.4)	67.6%(65.0-70.2)	8.6%(7.1-10.2)	31.9%(29.3-34.5)	31.7%(29.1-34.3)	26.9%(24.5-29.5)
P value for difference	＜0.0001	0.2331	0.1322	0.4401	＜0.0001	＜0.0001	0.1602	1.0000	0.0912	＜0.0001	＜0.0001	0.0394	0.1498	0.1174	＜0.0001
Hyperlipidemia															
Yes	29.9%(15.3-48.3)	4.8%(0.4-18.4)	25.1%(11.7-43.2)	41.8%(24.9-60.2)	65.1%(46.6-80.8)	26.5%(15.7-39.7)	18.0%(9.2-30.3)	32.4%(20.7-45.9)	33.3%(21.5-46.9)	56.4%(42.8-69.4)	30.1%(20.9-40.6)	12.9%(6.8-21.5)	26.8%(18-37.1)	34.8%(25.1-45.5)	62.2%(51.4-72.1)
No	73.5%(69.5-77.2)	7.0%(5.0-9.5)	25.7%(22.0-29.7)	27.8%(24-31.8)	32.7%(28.7-36.9)	64.1%(60.6-67.5)	7.9%(6.1-10.1)	35.6%(32.2-39.0)	35.1%(31.8-38.6)	23.9%(21.0-27.1)	68.0%(65.4-70.5)	7.4%(6-9)	31.7%(29.2-34.3)	32.1%(29.6-34.8)	27.4%(25.0-29.9)
P value for differe	＜0.0001	0.8903	1.0000	0.1255	0.0003	＜0.0001	0.0165	0.7301	0.8935	＜0.0001	＜0.0001	0.0943	0.3844	0.6806	＜0.0001

nce															
Hyperuricemia															
Yes	40.6%(19.1-65.2)	0.0%(0.0-17.6)	38.1%(17.2-62.8)	14.3%(2.7-37.8)	73.7%(48.8-90.8)	30.6%(13.0-53.6)	3.0%(0.0-20.5)	37.1%(17.8-60.1)	33.8%(15.3-56.9)	28.8%(11.8-51.8)	37.5%(22.9-54.0)	2.4%(0.1-12.8)	35.8%(21.5-52.3)	26.1%(13.7-42.2)	64.8%(48.3-79.0)
No	72.1%(68.1-75.8)	7.1%(5.1-9.6)	25.3%(21.6-29.2)	28.3%(24.5-32.3)	33.9%(29.9-38.1)	62.0%(58.6-65.4)	8.8%(6.9-11.0)	35.4%(32.1-38.8)	34.5%(31.2-37.9)	25.9%(22.9-29.1)	66.2%(63.6-68.8)	8.0%(6.6-9.6)	31.4%(28.9-34.0)	32.0%(29.5-34.5)	29.0%(26.6-31.5)
P value for difference	0.0067	0.4561	0.3221	0.2814	0.0009	0.0057	0.5724	1.0000	1.0000	0.9543	0.0003	0.3047	0.6681	0.5348	＜0.0001
Osteoarthritis of the knee															
Yes	34%(24.4-44.6)	0.0%(0.0-3.9)	17.9%(10.7-27.3)	45.5%(35.1-56.2)	69.3%(58.8-78.5)	25.7%(17.6-35.4)	7.5%(3.2-14.5)	27.2%(18.8-36.9)	53.1%(42.9-63.1)	53.2%(43.0-63.2)	30.8%(24.4-37.9)	4.0%(1.7-7.8)	22.9%(17.2-29.5)	47.1%(39.9-54.4)	61.8%(54.6-68.7)
No	77.4%(73.3-81.1)	8.2%(5.9-11.1)	27.8%(23.8-32.1)	25.4%(21.5-29.6)	27.9%(23.8-32.2)	67.2%(63.7-70.6)	8.8%(6.8-11.1)	36.5%(33.0-40.1)	31.8%(28.4-35.3)	22.4%(19.4-25.6)	71.2%(68.5-73.7)	8.5%(6.9-10.2)	33.2%(30.5-35.9)	29.3%(26.7-32.0)	24.6%(22.2-27.1)
P	＜	0.0088	0.0643	0.0002	＜	＜	0.8149	0.0837	＜	＜	＜	0.0451	0.0059	＜	＜

value for difference	0.0001				0.0001	0.0001			0.0001	0.0001	0.0001			0.0001	0.0001

Values are % (95% CI). Hypertension, diabetes, hyperlipidemia, hyperuricemia and osteoarthritis of the knee were defined as having been diagnosed by a physician in the past time. Smoker was defined as having smoked equal to or more than 100 cigarettes in the lifetime. According to the Chinese BMI index classification, BMI <18.5 kg/m^2 is malnutrition, 18.5 ≤ BMI < 24 kg/m^2 is normal, 24 ≤ BMI < 28 kg/m^2 is overweight, and ≥ 28 kg/m^2 is obesity.

Table S2. Age-specific and age-standardized prevalence of constipation among adults in the Urumqi city by nationality

	Han nationality					Other nationality					Total				
Age(Years)	Normal	Mild	Moderate	Severe	Very severe	Normal	Mild	Moderate	Severe	Very severe	Normal	Mild	Moderate	Severe	Very severe
20~29	34.7%(29.2-40.5)	3.1%(1.4-5.8)	24.7%(19.9-30.1)	21.6%(17.1-26.8)	15.8%(11.8-20.5)	36.4%(29.2-44.1)	5.8%(2.8-10.4)	12.7%(8.1-18.6)	22.5%(16.5-29.5)	22.5%(16.5-29.5)	35.3%(31-39.9)	4.1%(2.5-6.3)	20.3%(16.7-24.2)	22.0%(18.3-26)	18.3%(14.9-22.1)
30~39	27.7%(21.8-34.2)	5.2%(2.6-9.1)	22.5%(17.1-28.7)	22.1%(16.7-28.2)	22.5%(17.1-28.7)	29.2%(21.7-37.6)	4.4%(1.6-9.3)	16.1%(10.3-23.3)	24.1%(17.2-32.1)	26.3%(19.1-34.5)	28.3%(23.6-33.3)	4.9%(2.9-7.7)	20.0%(15.9-24.6)	22.9%(18.6-27.6)	24.0%(19.6-28.8)
40~49	35.8%(27.7-44.6)	3.0%(0.8-7.5)	20.1%(13.7-27.9)	19.4%(13.1-27.1)	21.6%(15.0-29.6)	19.7%(10.9-31.3)	3.0%(0.4-10.5)	22.7%(13.3-34.7)	31.8%(20.9-44.4)	22.7%(13.3-34.7)	30.5%(24.2-37.4)	3%(1.1-6.4)	21.0%(15.6-27.3)	23.5%(17.8-30.0)	22.0%(16.5-28.4)
50~59	49.2%(40.0-58.4)	4.9%(1.8-10.4)	20.5%(13.7-28.7)	15.6%(9.6-23.2)	9.8%(5.2-16.6)	39.2%(25.8-53.9)	3.9%(0.5-13.5)	17.6%(8.4-30.9)	15.7%(7.0-28.6)	23.5%(12.8-37.5)	46.2%(38.6-54)	4.6%(2.0-8.9)	19.7%(14.0-26.4)	15.6%(10.5-21.9)	13.9%(9.1-19.9)
≥60	60.2%(52.2-67	6.8%(3.5-11.	14.3%(9.3-20.	10.6%(6.3-16.	8.1%(4.4-13.4	36.8%(21.8-54	7.9%(1.7-21.4	15.8%(6.0-31.	21.1%(9.6-37.	18.4%(7.7-34.	55.8%(48.6-62	7.0%(3.9-11.	14.6%(10.0-20	12.6%(8.3-18.	10.1%(6.2-15.

	.9)	9)	7)	4)	)	)	)	3)	3)	3)	.8)	5)	.3)	0)	1)
P value for trend	< 0.0001	0.1136	0.0108	0.0018	0.0056	0.8062	0.9478	0.2477	0.7610	0.6967	< 0.0001	0.2203	0.1701	0.0028	0.0043
Gender															
Man	76.9%(72.3-81.1)	6.5%(4.3-9.5)	27.1%(22.7-31.9)	25.4%(21.1-30.1)	30.7%(26.1-35.6)	55.6%(48.0-63.1)	7.7%(4.3-12.7)	23.4%(17.3-30.3)	38.0%(30.9-45.6)	41.9%(34.5-49.5)	71.1%(67.2-74.9)	6.8%(4.9-9.3)	25.9%(22.3-29.7)	28.5%(24.8-32.5)	34.3%(30.4-38.4)
Women	63.9%(59.7-68.0)	8.7%(6.4-11.4)	38.9%(34.8-43.1)	32.8%(28.8-36.9)	22.4%(19.0-26.2)	52.8%(46.9-58.7)	9.1%(6.0-13.0)	31.2%(25.9-36.9)	37.9%(32.3-43.8)	35.6%(30.0-41.4)	61.2%(57.8-64.6)	8.8%(6.9-10.9)	35.9%(32.6-39.2)	34.6%(31.4-38.0)	26.2%(23.2-29.3)
P value for difference	< 0.0001	0.2838	0.0003	0.0196	0.0060	0.6227	0.7367	0.0847	1.0000	0.2054	0.0002	0.2301	0.0001	0.0207	0.0014
Urbanisation															
Urban	74.2%(71-77.3)	8.5%(6.6-10.6)	33.5%(30.1-36.9)	30.3%(27.1-33.6)	20.3%(17.5-23.3)	60.1%(54.6-65.3)	9.5%(6.5-13.1)	30.1%(25.3-35.4)	36.0%(30.8-41.4)	31.1%(26.1-36.3)	71.2%(68.4-73.8)	8.6%(7.0-10.4)	32.2%(29.4-35.0)	31.7%(28.9-34.5)	23.1%(20.6-25.7)
Rural	34.0%(25.6-43.1)	2.0%(0.3-6.3)	39.8%(31-49.1)	29.1%(21.2-38.1)	61.7%(52.5-70.4)	43.0%(33.8-52.6)	6.5%(2.7-12.7)	22.9%(15.5-31.7)	48.9%(39.4-58.4)	45.4%(36.1-55.0)	38.6%(32.3-45.1)	4.3%(2.1-7.8)	31.8%(25.9-38.2)	39.0%(32.7-45.6)	52.9%(46.3-59.4)
Subur	57.8%(	16.7%	33.3%(	12.2%(	46.7%(	9.5%(0	0.0%(0	11.1%(	10.3%(	68.7%(	31.2%(	11.1%	33.3%(	11.4%(	79.6%(

b	35.1-78.2)	(4.4-38.5)	15-56.4)	2.3-33.2)	25.4-68.9)	.8-33.6)	.0-19.5)	1.3-35.6)	1.0-34.6)	59.4-78.6)	17.4-48.0)	(3.3-25.3)	19.1-50.2)	3.5-25.7)	63.6-90.8)
P value for difference	＜0.0001	0.3794	0.3238	0.1718	＜0.0001	＜0.0001	0.1180	0.0330	0.6313	＜0.0001	＜0.0001	0.2160	0.9850	0.9406	＜0.0001
Education attainment															
Primary school and lower	59.4%(47.8-70.3)	5.8%(1.8-13.5)	19.9%(11.7-30.4)	28.7%(19.1-40.0)	52.9%(41.3-64.2)	49.8%(36-63.6)	6.5%(1.6-16.5)	11.1%(4.2-22.5)	48.1%(34.5-62.0)	51.1%(37.3-64.8)	54.0%(45.1-62.6)	7.0%(3.3-12.7)	16.2%(10.4-23.5)	39.4%(31.1-48.2)	50.2%(41.4-58.9)
Middle and high school	71.5%(65.2-77.2)	3.8%(1.7-7.1)	26.1%(20.6-32.2)	18.6%(13.8-24.2)	46.7%(40.2-53.3)	46.5%(36.8-56.4)	9.3%(4.6-16.4)	19.9%(12.9-28.7)	30.1%(21.7-39.7)	60.8%(50.9-70.1)	66.4%(61.2-71.4)	5.7%(3.5-8.7)	22.4%(18.1-27.2)	22.1%(17.8-26.9)	50%(44.6-55.4)
College and higher	61.7%(57.7-65.6)	11.5%(9.1-14.3)	37.5%(33.6-41.5)	33.5%(29.7-37.4)	22.5%(19.3-26.1)	60.8%(55.1-66.4)	10.8%(7.5-14.8)	34.3%(29.0-40.0)	36.4%(31.0-42.1)	24.4%(19.7-29.6)	61.1%(57.8-64.2)	11.1%(9.1-13.3)	36.4%(33.3-39.6)	34.3%(31.3-37.5)	23.8%(21-26.7)
P value for	0.2712	0.0019	0.0001	0.0037	＜0.0001	0.0189	0.3232	＜0.0001	0.3814	＜0.0001	0.7042	0.0085	＜0.0001	0.2631	＜0.0001

trend															
BMI															
Malnutrition	79.2%(68.5-87.6)	9.3%(3.9-18.1)	25.6%(16.4-36.8)	30.8%(20.8-42.2)	21.7%(13.2-32.5)	19.6%(9.7-33.2)	23.5%(12.7-37.6)	9.7%(3.2-21.4)	37.2%(23.9-52.0)	76.7%(62.6-87.5)	67.1%(58.3-75.2)	14.3%(8.8-21.6)	21.0%(14.3-29.1)	34.4%(26.3-43.3)	29.7%(22-38.5)
Normal	69.1%(65.2-72.9)	6.5%(4.6-8.9)	34.4%(30.5-38.4)	28.6%(24.9-32.5)	28.1%(24.4-31.9)	49.5%(43.5-55.5)	6.2%(3.7-9.8)	32.4%(26.9-38.2)	38.2%(32.5-44.2)	40.3%(34.5-46.3)	65.0%(61.7-68.2)	6.2%(4.7-8.1)	32.9%(29.7-36.1)	31.0%(27.9-34.3)	31.5%(28.4-34.8)
Overweight	69.2%(62.7-75.1)	8.6%(5.3-13.1)	34.6%(28.4-41.1)	32.7%(26.7-39.2)	21.5%(16.4-27.5)	57.7%(47.3-67.6)	12.3%(6.6-20.5)	27.9%(19.3-37.8)	33.6%(24.4-43.9)	35.2%(25.8-45.5)	66.0%(60.6-71.2)	9.6%(6.6-13.3)	32.4%(27.4-37.8)	33.0%(28.0-38.4)	25.6%(20.9-30.7)
Obesity	70.1%(54.8-82.7)	5.6%(1.0-16.6)	36.9%(23.2-52.4)	29.4%(16.9-44.7)	24.7%(13.2-39.6)	55.6%(38.6-71.6)	3.3%(0.2-14.9)	22.0%(10.3-38.4)	53.7%(36.8-70.0)	32.0%(17.9-49.1)	65.8%(54.6-75.8)	3.6%(0.7-10.1)	30.0%(20.4-40.9)	39.9%(29.4-51.2)	27.4%(18.3-38.3)
P value for trend	0.3375	0.9964	0.2619	0.5592	0.4106	0.0004	0.0719	0.5594	0.3425	0.0001	0.9901	0.1980	0.2582	0.3180	0.1505
Nature of work															
Office work	67.0%(61.4-72.3)	8.1%(5.3-11.8)	38.0%(32.5-43.7)	33.9%(28.6-39.5)	19.6%(15.3-24.5)	45.3%(37.5-53.4)	13.1%(8.3-19.4)	46.2%(38.3-54.3)	37.0%(29.5-44.9)	25.0%(18.5-32.4)	61.2%(56.6-65.7)	9.4%(6.9-12.5)	39.1%(34.6-43.7)	35.0%(30.7-39.6)	21.9%(18.2-25.9)
Manual	66.0%(60.5-71	8.1%(5.3-11.	30.1%(25.1-35	28.5%(23.6-33	33.9%(28.6-39	59.6%(51.4-67	8.8%(4.8-14.5	15.1%(9.9-21.	40.5%(32.6-48	42.6%(34.7-50	63.4%(58.9-67	8.3%(6.0-11.	25.8%(21.9-30	32.5%(28.3-37	36.5%(32.1-41

labor	.3)	7)	.5)	.9)	.4)	.5)	)	8)	.7)	.9)	.8)	2)	.1)	.0)	.1)
Outdoor work	51.2%(36.5-65.7)	6.7%(1.5-17.6)	6.7%(1.6-17.7)	27.3%(15.6-42.0)	74.7%(60.3-86.1)	32.3%(19.7-47.0)	8.3%(2.4-19.6)	6.3%(1.4-17.0)	31.3%(19.0-46.0)	88.5%(76.2-95.7)	43.2%(33.3-53.6)	8.2%(3.7-15.5)	7.2%(3.0-14.2)	27.8%(19.2-37.7)	80.3%(71.1-87.6)
Other	78.3%(72.8-83.2)	6.6%(3.9-10.4)	32.7%(27.0-38.9)	29.6%(24.0-35.6)	19.4%(14.8-24.8)	65.8%(55.7-74.9)	3.1%(0.7-8.6)	32.4%(23.4-42.3)	42.8%(33.1-53.0)	22.6%(14.9-31.9)	76.4%(71.7-80.8)	5.9%(3.7-8.9)	32.2%(27.4-37.3)	32.6%(27.8-37.8)	19.5%(15.5-24.0)
P value for difference	0.0071	0.4452	0.1042	0.3146	0.6851	0.0226	0.0067	0.0041	0.5302	0.1922	0.0001	0.0715	0.0107	0.3906	0.5747
Somker															
Yes	74.7%(67-81.4)	6.1%(2.9-11.2)	27.0%(20.1-34.9)	20.7%(14.5-28.0)	38.1%(30.3-46.4)	47.4%(34.7-60.4)	9.5%(3.6-19.6)	24.3%(14.4-36.8)	41.7%(29.4-54.8)	43.7%(31.2-56.8)	68.8%(62.2-75.0)	7.2%(4.1-11.5)	26.9%(21.1-33.4)	25.0%(19.3-31.4)	38.8%(32.2-45.6)
No	67.8%(64.3-71.0)	8.1%(6.3-10.2)	35.6%(32.2-39.1)	31.6%(28.3-35)	23.6%(20.7-26.8)	54.7%(49.7-59.7)	7.9%(5.5-11.0)	28.5%(24.1-33.2)	38.2%(33.4-43.1)	37.3%(32.6-42.3)	64.5%(61.7-67.3)	8.0%(6.5-9.7)	32.8%(30.1-35.5)	33.5%(30.8-36.3)	27.9%(25.3-30.5)
P value for difference	0.1103	0.5107	0.0532	0.0096	0.0003	0.3445	0.8542	0.5935	0.6937	0.4092	0.2539	0.7727	0.1073	0.0180	0.0018
Daily															

water intake															
＜1000 mL	57.0%(52.4-61.4)	7.2%(5.0-9.9)	31.4%(27.2-35.7)	34.8%(30.5-39.2)	36.3%(32.0-40.8)	45.3%(39.3-51.4)	7.6%(4.8-11.4)	26.7%(21.5-32.3)	36.5%(30.8-42.5)	50.6%(44.5-56.7)	54.0%(50.4-57.6)	7.2%(5.5-9.3)	29.5%(26.3-32.9)	34.8%(31.4-38.3)	41.1%(37.5-44.7)
⩾1000 mL	82.9%(79.1-86.3)	8.1%(5.8-11.1)	36.9%(32.4-41.6)	24.3%(20.4-28.6)	14.4%(11.3-18)	66.1%(58.9-72.8)	9.2%(5.5-14.2)	30.9%(24.4-38.0)	40.9%(33.9-48.2)	19.6%(14.2-25.9)	79.1%(75.7-82.2)	8.6%(6.6-11.1)	34.6%(30.9-38.4)	28.8%(25.3-32.5)	15.5%(12.8-18.6)
P value for difference	＜0.0001	0.6764	0.0903	0.0007	＜0.0001	＜0.0001	0.6630	0.3757	0.3830	＜0.0001	＜0.0001	0.3793	0.0505	0.0195	＜0.0001
Daily salt consumption															
＜6g	79.4%(70.0-86.9)	7.6%(3.2-14.8)	44.1%(34.0-54.6)	17.8%(10.7-26.8)	17.8%(10.8-26.9)	64.6%(51.8-76.0)	16.0%(8.1-27.0)	28.4%(18.0-40.9)	40.4%(28.5-53.2)	17.3%(9.1-28.6)	76.0%(68.6-82.3)	10.7%(6.4-16.5)	38.1%(30.6-46.0)	25.6%(19.1-33.1)	16.2%(10.9-22.8)
⩾6g	68.2%(64.9-71.3)	7.6%(5.9-9.7)	32.3%(29.1-35.6)	31.4%(28.2-34.7)	27.2%(24.2-30.4)	53.6%(48.5-58.5)	6.0%(3.9-8.8)	28.6%(24.3-33.3)	36.6%(31.9-41.6)	41.8%(37.0-46.9)	64.4%(61.7-67.1)	7.3%(5.9-8.9)	30.5%(27.9-33.2)	33.0%(30.4-35.7)	31.4%(28.8-34.1)
P value	0.0315	1.0000	0.0268	0.0080	0.0616	0.1247	0.0093	1.0000	0.6549	0.0003	0.0048	0.1758	0.0599	0.0717	0.0001

for difference															
Hours of physical activity per week															
＜1h	67.6%(63.1-71.8)	7.2%(5.1-10.0)	37.8%(33.4-42.4)	33.6%(29.3-38.1)	20.5%(16.9-24.4)	51.6%(45.1-58.0)	7.4%(4.5-11.4)	37.9%(31.8-44.3)	39.8%(33.6-46.2)	29.9%(24.3-36.1)	62.6%(58.9-66.2)	7.3%(5.5-9.5)	37.7%(34.1-41.4)	35.4%(31.9-39.0)	23.7%(20.6-2.07)
1~2.5h	66.8%(61.2-72.1)	6.5%(4.0-9.9)	29.9%(24.9-35.4)	28.4%(23.4-33.8)	35.0%(29.6-40.6)	46.6%(38.5-54.8)	8.8%(4.8-14.4)	16.7%(11.2-23.6)	44.9%(36.9-53.1)	49.6%(41.5-57.8)	61.9%(57.3-66.4)	7.3%(5.1-10.0)	24.8%(20.9-29.0)	33.6%(29.3-38.1)	39.1%(34.6-43.7)
⩾2.5h	82.4%(75.3-88.1)	11.3%(6.7-17.4)	28.0%(20.9-35.9)	19.1%(13.1-26.3)	25.9%(19.1-33.7)	76.2%(64.1-85.8)	13.9%(6.6-24.6)	21.1%(12.0-32.9)	17.2%(9.0-28.5)	38.3%(26.6-51.1)	81.4%(75.5-86.3)	11.1%(7.2-16.1)	25.3%(19.7-31.7)	18.7%(13.7-24.5)	30.1%(24.1-36.7)
P value for trend	0.0049	0.2181	0.0084	0.0007	0.0081	0.0118	0.1274	0.0001	0.0199	0.0103	＜0.0001	0.1342	＜0.0001	＜0.0001	0.0004
Sleep disorder															

Yes	47.6%(41.7-53.5)	6.5%(3.9-10.0)	36.3%(30.7-42.2)	34.3%(28.8-40.1)	42.0%(36.3-48.0)	44.3%(36.0-53.0)	5.9%(2.6-11.2)	27.2%(20.0-35.4)	37.6%(29.5-46.1)	51.6%(43.0-60.2)	46.7%(41.9-51.6)	6.2%(4.1-8.9)	33.9%(29.4-38.6)	35.0%(30.4-39.7)	44.9%(40.1-49.8)
No	78.7%(75.3-81.9)	7.8%(5.9-10.2)	33.3%(29.7-37.1)	28.0%(24.6-31.7)	18.7%(15.8-22.0)	58.9%(53.3-64.3)	8.8%(6.0-12.4)	29.6%(24.7-34.9)	38.3%(32.9-43.8)	31.2%(26.2-36.5)	73.8%(70.9-76.6)	8.3%(6.6-10.2)	31.2%(28.2-34.2)	31.1%(28.2-34.2)	22.3%(19.7-25.1)
P value for difference	<0.0001	0.5469	0.4226	0.0651	<0.0001	0.0054	0.3832	0.6878	0.9720	<0.0001	<0.0001	0.2197	0.3397	0.1797	<0.0001
Regularity in one's daily life															
Yes	78.6%(74.7-82.1)	7.9%(5.7-10.7)	34.4%(30.2-38.7)	22.6%(19.0-26.5)	23.2%(19.6-27.2)	57.2%(50.8-63.4)	8.1%(5.0-12.2)	29.8%(24.2-35.9)	44.3%(38.1-50.7)	27.3%(21.8-33.2)	73.3%(69.9-76.4)	8.1%(6.2-10.3)	32.0%(28.7-35.5)	28.9%(25.6-32.2)	24.5%(21.4-27.7)
No	54.8%(49.9-59.6)	5.6%(3.6-8.2)	30.5%(26.1-35.2)	42.5%(37.7-47.4)	33.3%(28.8-38.0)	47.3%(40.5-54.2)	7.5%(4.4-11.9)	23.4%(17.9-29.6)	31.5%(25.4-38.1)	56.9%(50.0-63.6)	52.7%(48.7-56.6)	6.3%(4.5-8.4)	28.3%(24.9-32.0)	39.2%(35.4-43.1)	40.2%(36.3-44.1)
P value for	<0.0001	0.2014	0.2417	<0.0001	0.0009	0.0422	0.9529	0.1484	0.0060	<0.0001	<0.0001	0.2322	0.1529	0.0001	<0.0001

difference															
Hypertension															
Yes	49.4%(41.8-57.0)	5.7%(2.8-10.2)	28.4%(21.9-35.7)	22.4%(16.5-29.2)	60.8%(53.2-68.1)	28.1%(18.4-39.5)	3.7%(0.7-10.7)	25.6%(16.3-36.8)	36.0%(25.4-47.8)	73.3%(61.9-82.7)	43.0%(36.9-49.4)	5.3%(2.9-8.8)	27.2%(21.9-33.2)	27.0%(21.7-32.9)	64.1%(57.9-70.0)
No	75.5%(72.2-78.5)	7.9%(6.0-10.0)	32.8%(29.5-36.3)	30.7%(27.4-34.2)	19.8%(17.0-22.8)	59.3%(54.2-64.2)	9.7%(6.9-13.1)	27.9%(23.5-32.7)	39.8%(34.9-44.8)	30.0%(25.4-34.8)	71.5%(68.7-74.1)	8.3%(6.8-10.1)	30.8%(28.1-33.6)	33.1%(30.3-35.9)	23.0%(20.6-25.5)
P value for difference	＜0.0001	0.4030	0.2985	0.0359	＜0.0001	＜0.0001	0.1399	0.7811	0.6232	＜0.0001	＜0.0001	0.1268	0.2973	0.0733	＜0.0001
Diabetes															
Yes	47.6%(35.5-60.0)	1.2%(0.0-7.4)	24.3%(14.8-36.2)	42.8%(31.0-55.3)	50.7%(38.4-62.9)	26.4%(14.7-41.1)	2.6%(0.1-11.8)	26.0%(14.4-40.6)	36.6%(23.2-51.8)	75.1%(60.5-86.4)	38.7%(29.9-48.2)	2.7%(0.6-7.5)	24.9%(17.4-33.8)	39.2%(30.3-48.7)	61.1%(51.6-70.0)
No	70.9%(67.7-73.9)	8.3%(6.5-10.3)	34.2%(31.0-37.5)	28.8%(25.8-32.0)	24.5%(21.6-27.5)	56.5%(51.6-61.3)	9.4%(6.8-12.7)	28.0%(23.8-32.6)	39.6%(34.9-44.5)	33.1%(28.6-37.8)	67.6%(65.0-70.2)	8.6%(7.1-10.2)	31.9%(29.3-34.5)	31.7%(29.1-34.3)	26.9%(24.5-29.5)
P value	0.0001	0.0596	0.1248	0.0210	＜0.0001	0.0001	0.1838	0.8948	0.8114	＜0.0001	＜0.0001	0.0394	0.1498	0.1174	＜0.0001

for difference															
Hyperlipidemia															
Yes	32.1%(20.4-45.8)	12.3%(5.1-23.7)	20.5%(10.9-33.3)	48.6%(35.2-62.2)	53.1%(39.5-66.5)	25.5%(12.2-43.3)	7.9%(1.4-22.4)	38.8%(22.7-57.0)	19.3%(7.8-36.4)	75.2%(57.4-88.3)	30.1%(20.9-40.6)	12.9%(6.8-21.5)	26.8%(18.0-37.1)	34.8%(25.1-45.5)	62.2%(51.4-72.1)
No	71.8%(68.7-74.8)	7.1%(5.5-9.1)	34.6%(31.4-37.9)	28.8%(25.8-31.9)	24.3%(21.5-27.3)	56.1%(51.2-60.8)	8.4%(6.0-11.5)	26.8%(22.7-31.3)	40.4%(35.8-45.2)	34.9%(30.4-39.6)	68.0%(65.4-70.5)	7.4%(6.0-9.0)	31.7%(29.2-34.3)	32.1%(29.6-34.8)	27.4%(25.0-29.9)
P value for difference	＜0.0001	0.2396	0.0413	0.0026	＜0.0001	0.0011	1.0000	0.1912	0.0243	＜0.0001	＜0.0001	0.0943	0.3844	0.6806	＜0.0001
Hyperuricemia															
Yes	45.6%(27.1-65.0)	4.2%(0.2-18.9)	44.7%(26.4-64.2)	17.5%(6.0-36.1)	54.7%(35.3-73.2)	16.7%(2.1-48.4)	0.0%(0.0-26.5)	11.1%(0.6-42.0)	33.3%(9.9-65.1)	72.2%(40.1-93.1)	37.5%(22.9-54.0)	2.4%(0.1-12.8)	35.8%(21.5-52.3)	26.1%(13.7-42.2)	64.8%(48.3-79.0)
No	70.2%(67.1-73.2)	7.8%(6.1-9.7)	33.4%(30.3-36.6)	29.5%(26.5-32.6)	25.8%(22.9-28.8)	54.4%(49.7-59.1)	8.5%(6.1-11.5)	28.5%(24.4-32.9)	38.4%(33.9-43)	36.9%(32.5-41.6)	66.2%(63.6-68.8)	8.0%(6.6-9.6)	31.4%(28.9-34.0)	32.0%(29.5-34.5)	29.0%(26.6-31.5)

P value for difference	0.0085	0.7148	0.2833	0.2324	0.0011	0.0219	0.6007	0.3190	0.9579	0.0288	0.0003	0.3047	0.6681	0.5348	＜ 0.0001
Osteoarthritis of the knee															
Yes	39.1%(29.8-49.1)	4.2%(1.2-10.0)	28.4%(20.0-38.0)	39.2%(29.8-49.2)	55.8%(45.8-65.5)	22.6%(14.4-32.8)	4.2%(1.1-10.7)	15.6%(8.7-24.8)	57.7%(46.7-68.1)	66.7%(55.8-76.3)	30.8%(24.4-37.9)	4.0%(1.7-7.8)	22.9%(17.2-29.5)	47.1%(39.9-54.4)	61.8%(54.6-68.7)
No	73.0%(69.8-76.1)	8.0%(6.2-10.1)	34.7%(31.4-38.1)	28.4%(25.3-31.6)	22.6%(19.7-25.6)	62.1%(57.0-67.0)	9.5%(6.8-13)	32.2%(27.5-37.1)	31.6%(26.9-36.6)	31.3%(26.6-36.2)	71.2%(68.5-73.7)	8.5%(6.9-10.2)	33.2%(30.5-35.9)	29.3%(26.7-32.0)	24.6%(22.2-27.1)
P value for difference	＜ 0.0001	0.2305	0.2397	0.0302	＜ 0.0001	＜ 0.0001	0.1565	0.0031	＜ 0.0001	＜ 0.0001	＜ 0.0001	0.0451	0.0059	＜ 0.0001	＜ 0.0001

Values are % (95% CI). Hypertension, diabetes, hyperlipidemia, hyperuricemia and osteoarthritis of the knee were defined as having been diagnosed by a physician in the past time. Smoker was defined as having smoked equal to or more than 100 cigarettes in the lifetime. According to the Chinese BMI index classification, BMI <18.5 kg/m^2 is malnutrition, 18.5 ≤ BMI < 24 kg/m^2 is normal, 24 ≤ BMI < 28 kg/m^2 is overweight, and ≥ 28 kg/m^2 is obesity.

Table S3. Dietary factors and factor loadings

Dietary patterns	Factor loadings	Eigenvalue	Variance contribution (%)	Cumulative variance contribution (%)
Dietary pattern 1		5.40	21.58	21.85
White wine	0.84			
Yellow wine	0.81			
Sweets, snacks, cakes	0.65			
Beer	0.62			
Dietary pattern 2		2.34	9.35	30.93
Red meat dishes (e.g. pork, beef, lamb)	0.71			
Poultry dishes (e.g. chicken, duck, goose)	0.90			
Eggs or duck eggs	0.66			
Dietary pattern 3		1.44	5.76	36.69
Coarse grains (including brown rice, millet, corn, barley, oats, red beans, mung beans, etc.)	0.71			
Potatoes (including sweet potatoes, potatoes, taro, yams, konjac, etc.)	0.62			
Dietary pattern 4		1.31	5.25	41.93
Dark-colored vegetable dishes (e.g., bok choy, spinach, cabbage, tomatoes, peppers, carrots, etc.)	0.74			
Light-colored vegetables (e.g. cabbage, radish, cucumber, etc.)	0.72			
Dietary pattern 5		1.26	5.03	46.96
Seafood dishes (scallop, pomfret,	0.70			

yellowtail, sea shrimp, etc.)				
Dietary patterns 6		1.10	4.42	51.38
Rice	0.67			

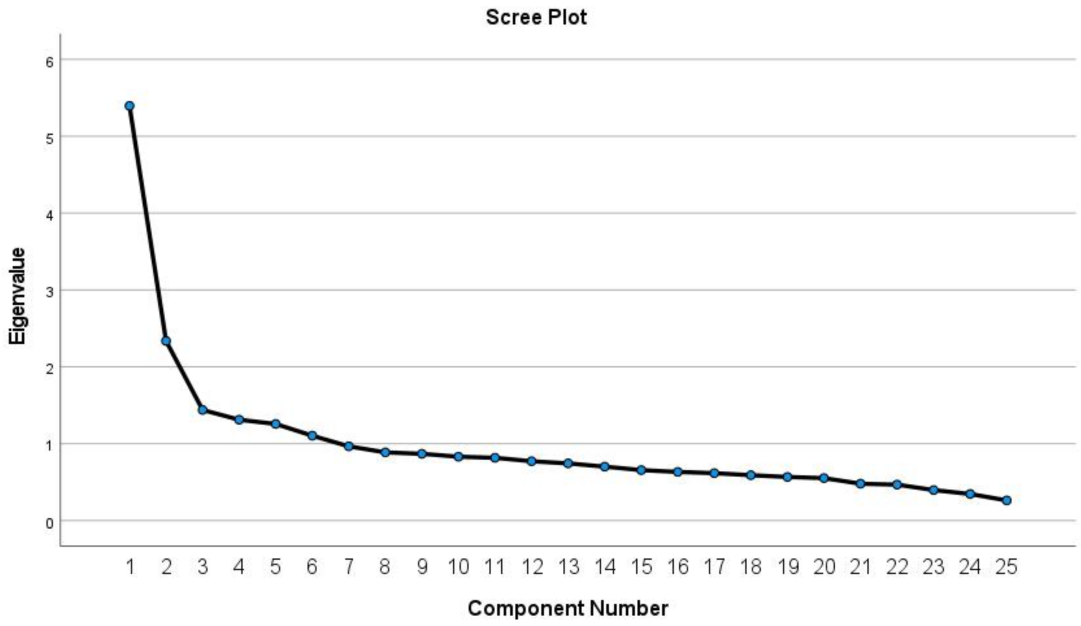

Figure S2. Scree Plot

Table S4. Multiple linear regression analysis of PAC-SYM total scores of adults in Urumqi city by gender and nationality

	Man		Women		Han nationality		Other nationality	
	β (95%CI)	P	β (95%CI)	P	β (95%CI)	P	β (95%CI)	P

Constants	7.31(-0.28~14.88)	0.0591	8.39(2.92~13.86)	0.0027	10.89(4.97~16.80)	0.0003	13.03(5.12~20.9)	0.0013
Women	-	-	-	-	0.18(-1.11~1.47)	0.7844	-0.85(-2.84~1.13)	0.3975
Age(10 year increments)	-1.69(-2.37-1.02)	＜0.0001	-0.26(-0.85~0.34)	0.3957	-0.09(-0.14~-0.04)	0.0003	-0.97(-1.80~-0.14)	0.0222
Other nationality	0.57(-1.16~2.29)	0.5177	0.52(-0.74~1.78)	0.4191	-	-	-	-
Rural resident	2.27(0.13~4.42)	0.0381	1.94(0.20~3.68)	0.0292	3.20(1.46~4.94)	0.0003	0.10(-2.09~2.29)	0.9267
Suburb residents	3.01(-1.56~7.59)	0.1960	6.52(2.64~10.40)	0.0010	4.40(0.7~8.05)	0.0185	2.37(-2.94~7.69)	0.3809
Education attainment								
Primary school and lower	0.00		0.00		0.00		0.00	
Middle and high school	1.75(-0.97~4.47)	0.2062	-0.38(-2.73~1.98)	0.7540	0.03(-2.18~2.23)	0.9814	0.49(-2.68~3.66)	0.7606
College and higher	-2.14(-4.90~0.63)	0.1291	0.10(-2.37~2.58)	0.9347	-1.67(-4.01~0.66)	0.1592	-0.95(-4.01~2.11)	0.5422
BMI								
Underweight	-1.95(-6.04~2.13)	0.3481	-0.74(-2.53~1.06)	0.4214	-0.19(-2.22~1.83)	0.8510	-1.82(-4.78~1.14)	0.2269
Normal	0.00		0.00				0.00	
Overweight	-1.31(-3.07~0.46)	0.1474	-0.84(-2.37~0.68)	0.2772	-1.39(-2.73~-0.05)	0.0426	-0.78(-3.00~1.45)	0.4938
Obesity	-0.20(-3.31~2.90)	0.8978	-2.79(-5.50~-0.09)	0.0432	-1.60(-4.24~1.04)	0.2335	-2.07(-5.40~1.25)	0.2212
Nature of work								
Office work	0.00		0.00		0.00		0.00	
Manual labor	0.13(-1.84~2.10)	0.8990	0.63(-0.81~2.08)	0.3902	0.70(-0.70~2.09)	0.3284	0.21(-1.94~2.36)	0.8489
Outdoor work	1.70(-1.26~4.65)	0.2599	3.88(0.81~6.94)	0.0134	3.51(0.81~6.22)	0.0110	1.13(-2.35~4.61)	0.5237
Other	-2.13(-4.26~-0.01)	0.0495	-0.62(-2.18~0.94)	0.4344	-0.91(-2.38~0.57)	0.2281	-0.85(-3.27~1.56)	0.4870
Smoker	0.11(-1.55~1.77)	0.8942	2.16(-1.14~5.45)	0.1993	0.52(-1.14~2.18)	0.5425	-0.09(-2.88~2.70)	0.9509
Daily water intake ≥1000mL/day	-2.03(-3.71~-0.35)	0.0180	-2.31(-3.52~-1.09)	0.0002	-2.19(-3.35~-1.02)	0.0003	-3.11(-4.97~-1.25)	0.0011
Daily salt consumption ≥ 6g	2.84(0.21~5.47)	0.0343	0.78(-0.97~2.52)	0.3843	1.14(-0.69~2.97)	0.2217	1.45(-1.08~3.98)	0.2603
Hours of physical activity per week								
＜1h	0.00		0.00		0.00		0.00	
1~2.5h	2.26(0.48~4.04)	0.0131	0.50(-0.86~1.86)	0.4728	1.83(0.55~3.11)	0.0050	0.88(-1.19~2.96)	0.4038

≥2.5h	1.44(-0.74~3.62)	0.1962	0.50(-1.37~2.36)	0.6027	0.97(-0.67~2.60)	0.2464	0.28(-2.43~2.99)	0.8379
Sleep disorder	3.21(1.42~4.99)	0.0005	2.42(1.13~3.71)	0.0002	2.96(1.7~4.19)	＜0.0001	1.83(-0.19~3.84)	0.0751
Regularity in one's daily life	-1.34(-2.97~0.30)	0.1088	-2.30(-3.55~-1.05)	0.0003	-2.06(-3.25~-0.87)	0.0007	-2.33(-4.16~-0.51)	0.0124
Hypertension	3.79(1.80~5.77)	0.0002	2.61(0.75~4.48)	0.0061	3.73(2.16~5.30)	＜0.0001	4.22(1.61~6.83)	0.0016
Diabetes	2.01(-0.47~4.49)	0.1116	1.44(-1.18~4.06)	0.2807	0.38(-1.84~2.60)	0.7367	3.66(0.54~6.78)	0.0216
Hyperlipidemia	2.76(-0.64~6.16)	0.1109	1.52(-0.89~3.93)	0.2151	3.01(0.65~5.37)	0.0126	1.06(-2.57~4.70)	0.5663
Hyperuricemia	-1.81(-6.13~2.51)	0.4105	-1.24(-4.94~2.47)	0.5131	-2.00(-5.27~1.26)	0.2293	0.23(-5.55~6.01)	0.9376
Osteoarthritis of the knee	4.76(2.50~7.02)	＜0.0001	3.33(1.39~5.27)	0.0008	2.74(0.85~4.63)	0.0046	6.66(4.24~9.08)	0
Dietary pattern 1	0.58(-0.07~1.23)	0.0797	2.43(1.54~3.32)	＜0.0001	1.29(0.70~1.88)	＜0.0001	0.86(-0.10~1.82)	0.0801
Dietary pattern 2	-1.20(-1.87~-0.52)	0.0005	-0.08(-0.79~0.63)	0.8241	-0.82(-1.40~-0.25)	0.0053	-0.46(-1.32~0.41)	0.3005
Dietary pattern 3	-0.58(-1.34~0.19)	0.1416	-0.11(-0.72~0.50)	0.7240	-0.74(-1.34~-0.13)	0.0182	0.28(-0.55~1.11)	0.5017
Dietary pattern 4	-0.48(-1.22~0.27)	0.2068	-0.54(-1.25~0.16)	0.1308	-0.53(-1.15~0.08)	0.0879	-0.71(-1.61~0.20)	0.1242
Dietary pattern 5	1.39(0.71~2.07)	0.0001	0.61(-0.12~1.35)	0.1024	1.19(0.61~1.77)	0.0001	1.20(0.28~2.12)	0.0108
Dietary pattern 6	-1.50(-2.18~-0.81)	＜0.0001	-0.45(-1.17~0.26)	0.2117	-0.77(-1.33~-0.21)	0.0071	-1.51(-2.47~-0.56)	0.0020
